# The intra- and inter-rater reproducibility of bone level measurements at strategic mini-implants using dental panoramic radiography

**DOI:** 10.1016/j.clinsp.2023.100316

**Published:** 2023-12-12

**Authors:** Martin Strauch, Ahmad Al Jaghsi, Christian Schwahn, Torsten Mundt

**Affiliations:** a Department of Prosthodontics, Gerodontology and Dental Materials, Greifswald University Medicine, Germany; b Clinical Sciences Department, College of Dentistry, Ajman, United Arab Emirates; c Centre of Medical and Bio-Allied Health, Sciences Research, Ajman University, Ajman, United Arab Emirates

**Keywords:** Dental implants, Alveolar bone loss, Panoramic radiography, Reproducibility of results, Correlation of data

## Abstract

**Objectives:**

This experimental study focused on the intra- and inter-rater reproducibility of vertical bone level (VBL) measurements at strategic mini-implants (MI) using digital panoramic radiographs (PR).

**Study design:**

VBLs of 152 MIs for removable partial denture stabilization at 50 randomly chosen PRs from a clinical trial were digitally evaluated by three ratters. Rater deviations exceeding 0.5 mm were re-examined. The intra-class correlation coefficient (ICC) was applied to estimate reliability. The smallest detectable change (SDC) was interrelated to the minimal clinically important change of 0.2 mm.

**Results:**

The first measurement round revealed intra- and inter-rater ICCs of > 0.8. However, 28 sites (9 %) were unreadable, and 97 sites (32 %) revealed differences between observers of ≥ 0.5 mm. Following a consensus session and re-training, an additional 8 sites were excluded and all remaining VBL differences were ≤ 0.5 mm. Thus, the SDCs with 95 % credibility were improved from 0.73 to 0.31 mm in the intra-rater and from 1.52 to 0.34 mm in the inter-rater statistics. Given a 50 % credibility for this special setting, both the intra- and inter-rater SDCs were 0.11 mm.

**Conclusions:**

Digital PR can be reliably utilized to determine VBLs around MIs under conditions of at least two trained observers, mutual calibration sessions, and exclusion of unquantifiable radiographs.

**German Clinical Trials Register ID:**

DRKS00007589, www.germanctr.de

## Highlights

• Panoramic radiography is suitable for determining vertical bone loss around mini-implants.
• Questionable implant sites are to be evaluated in cooperation with one other expert.
• Unquantifiable implant sites are to be excluded regarding therapy decisions.
• Mutual calibration sessions for bone level measurements are highly recommended.

**Statement of clinical relevance**

Digital panoramic radiography should be carefully used to determine vertical bone loss around mini-implants. Questionable implant sites are to be rated in cooperation with one other expert or should be consequently excluded regarding therapy decisions.

Alt-text: Unlabelled box

## 1 Introduction

One basic success criterion besides clinical parameters in implant dentistry is the radiographic Vertical Bone Level (VBL) change around dental implants.^1^, ^2^, ^3^ A radiographic bone loss up to 1.5‒2 mm in the first year after initial surgery is usually rated as remodeling of the bone and not necessarily as unphysiological peri‑implantitis.^2^, ^3^, ^4^ Albrektsson et al. defined radiographic implant success as a mean bone loss of ≤ 0.2 mm following the first year of service.^1^ VBL measurements at mesial and distal implant sites using X-rays are not only used by practitioners to monitor VBL during maintenance care but also for longitudinal evaluations of treatment methods or implant surfaces and designs.^2^^,^^4^

Radiographic images should be of high quality to detect the bone-to-implant border and reference points, e.g., the implant shoulder.^5^ Implants on 2-dimensional radiographs can be foreshortened or enlarged due to the imaging technique, the imaging plane relative to the bone, and/or implant angulation within the bone. Therefore, real implant lengths, distances between screw threads or reference markers, e.g., steel balls, are used for the image calibration.^6^, ^7^, ^8^ The gold standard for VBL measurements is comparable Intraoral Radiographs (IR) with repositionable keys like acrylic bite blocks or splints to ensure the reproducible angulation of the film-holding device.^4^^,^^7^ IRs are superior to Panoramic Radiography (PR) due to higher resolution, lower distortion, fewer anatomic superimpositions, and lower radiation dose.^6^^,^^9^ The mean enlargement of IRs is < 10 % is lower than that of PR at about 20 %.^7^^,^^10^ With regards to the precision and validity of IR, even IR measurements can underestimate the intra-operatively real bone loss at implants by up to several millimeters.^11^, ^12^, ^13^ It is sometimes difficult to place the intraoral film, owing to the resilient mouth floor, anatomical features, or the urge to gag in posterior regions.^6^ Digital PR have a relatively low-dose radiation in the case of multiple implants per person and is simple to perform without any additional devices.^4^^,^^6^^,^^7^^,^^14^ However, PR can produce artifacts, because of the semicircular imaging functionality that leads to superimpositions of anatomic structures.^10^ Nevertheless, in the assessment of marginal bone height at teeth and implants, PR was found to be as reliable or only slightly poorer as at intraoral radiographs.^4^^,^^6^^,^^7^^,^^14^, ^15^, ^16^ An individual rater of radiographs could be biased. Therefore, several raters are recommended, and repeated measurements of one rater should be averaged.^6^^,^^14^^,^^17^ In the case of large discrepancies, the observers should reach a consensus regarding the correct measuring point. Implant sites that cannot be reliably assessed must be excluded from further analyses.^14^ Measurement reproducibility includes the concepts of agreement and reliability which are related to comparisons between groups and within subjects, respectively.^18^ Agreement parameters estimate the exact differences and the measurement error in units of measurement and reflect the performance of the measurement instrument to detect clinically important changes. According to the methodological literature, the proposed limit of ≤ 0.2 mm bone loss at implants per year following the first year of service would be the Minimal Clinically Important Change (MCIC) similar to patient-centered outcomes.^19^^,^^20^ Despite good to excellent reliability in terms of unitless Intra-Class Correlation Coefficients (ICC) of > 0.8, the agreement for VBL measurements is questionable given the MCIC of 0.2 mm.^12^^,^^13^^,^^21^, ^22^, ^23^, ^24^, ^25^ In former studies, both mean intra- and inter-rater differences were between 0.3 and 0.5 mm with standard deviations of > 0.5 mm.^21^^,^^22^^,^^26^^,^^27^ Until now it is unknown whether the agreement in terms of the Smallest Detectable Change (SDC) is smaller than the MCIC of 0.2 mm, representing an acceptable level of agreement in units of measurement.^19^

In a 3-year, multi-center, randomized clinical trial, strategic mini-implants were placed for the stabilization of Removable Partial Dentures (RPDs) and either immediately or delayed loaded.^28^ The primary outcome was bone level change. This experimental study focused on the intra- and inter-rater reliability in terms of ICC and observer differences as well as the agreement of the VBL measurements in terms of Standard Error of Measurement (SEM) and SDC.

## 2 Materials and methods

### 2.1 Patient and material selection

**Fig. 1:**
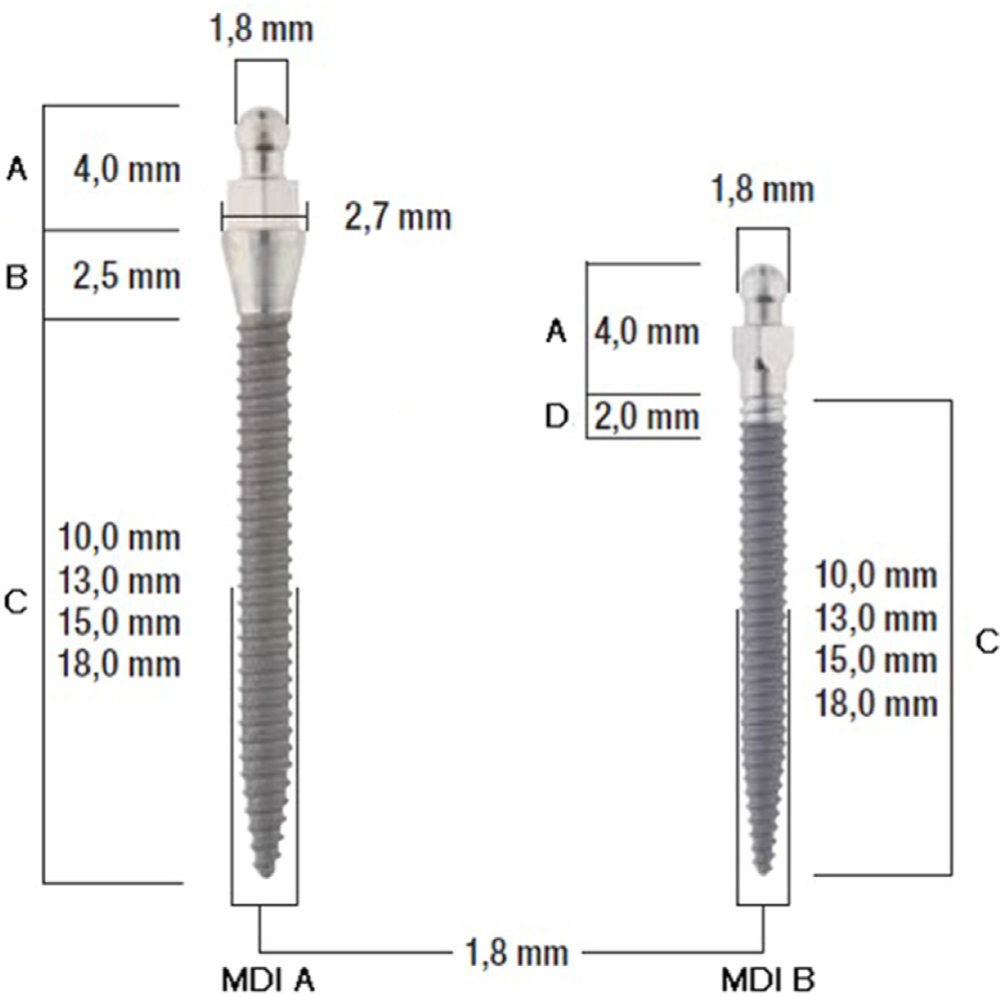
Types of mini-implants (MDI A collared, MDI B without collar), Distance: (A) Ball abutment with insertion square; (B) Polished collar; (C) Thread length; (D) Polished thread part.

**Fig. 2:**
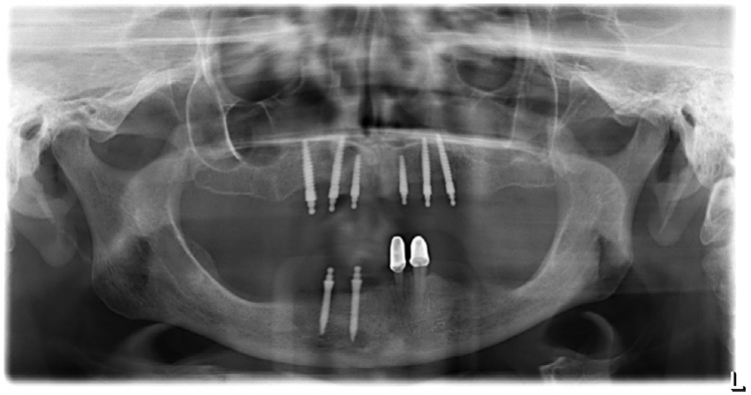
Panoramic radiography image with various mini-implants.

The study protocol was approved by the Ethics Committee of the University of Greifswald, Germany (approval number: BB 058/13A). The images were obtained from a multicenter randomized clinical trial registered in the German Clinical Trials Register system (Deutsches Register Klinischer Studien, DRKS-ID: DRKS00007589, www.germanctr.de). A total of 232 one-piece MIs (Mini Dental Implant, MDI, formerly manufactured by 3 M ESPE, Seefeld, Germany, and now by Condent, Hannover, Germany) were placed in 31 maxillary and 48 mandibular jaws among 76 participants in a university hospital and in three dental practices. The MIs had intraosseous screw length of 10, 13, or 15 mm and diameters of 1.8, 2.1, or 2.4 mm (Fig. 1). For this study 50 panoramic radiographs from the 31 participants of the university were randomly selected (Fig. 2). The standardized digital panoramic radiographs were taken post-surgery and at follow-ups after one year and three years with Orthophos XG Plus (Sirona Dental Systems GmbH, 2006 Bensheim, Germany) and a CEI OCX 100 tube using a voltage setting of 60‒90 kV at 12 mA and 15 s exposure time. The X-ray image was automatically transmitted to the installed computer program (SIDEXIS XG 2.61, Sirona Dental Systems GmbH, Bensheim, Germany) of the university data pool, exported in a separate folder and saved with a resolution of 235 dpi in a file.tif format.

### 2.2 Radiographic measurement

**Fig. 3:**
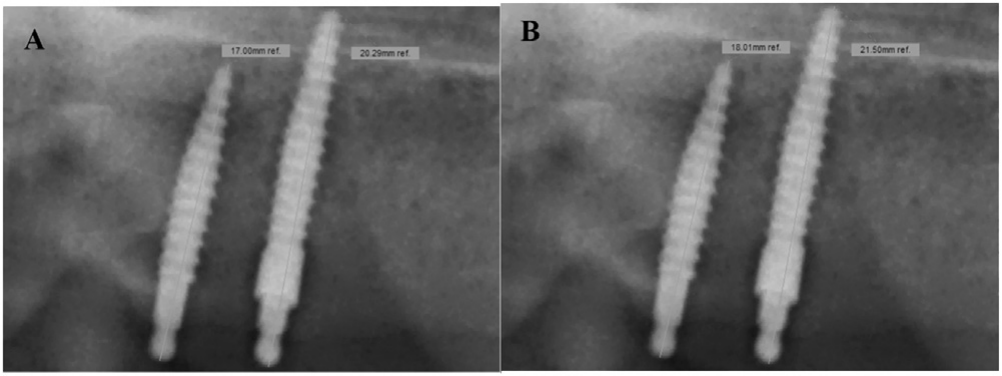
Calibration to the real implant length for a uncollared mini-implant (left side 3A), screw length 13 mm, after correction at the left radiograph) and a collared mini-implant (right side 3B), screw length 15 mm, after correction at the right radiograph).

**Fig. 4:**
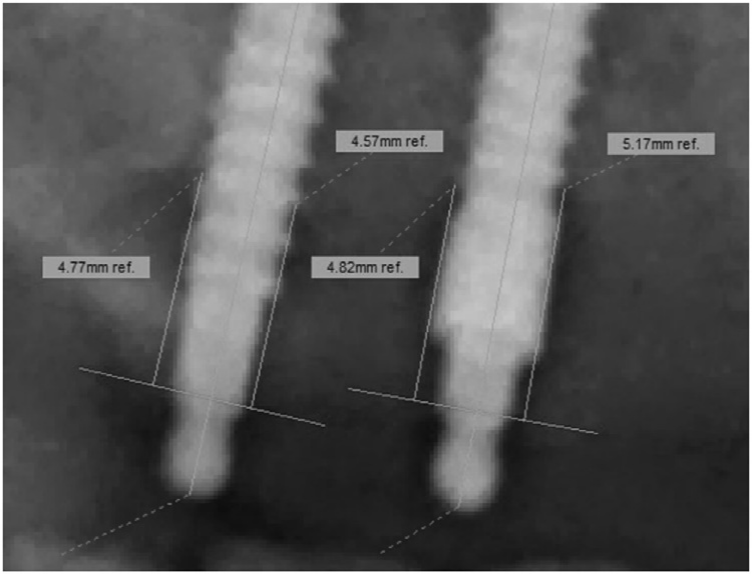
Aid lines and distances (mm) after length calibration between insertion square and first visible bone-to-implant contact.

Prior to the analyses, a standard operating procedure was designed and explained in training sessions by experienced dentists to calibrate the examiners. All measurements were performed by three observers: two research assistants (Rater 1 and 3) and one radiologist (Rater 2). Rater 1 assessed the data twice at a distance of four weeks to estimate the intra-rater reliability. The radiographs were analyzed on a high-resolution 23″ screen monitor, approved for radiology diagnoses (Dell, HD 1.920×1.080 pixel). Images of unsatisfactory quality, e.g., vague bone borders, or blurred implants that were out of focus were excluded in concert with the supervisor. By using the digital calliper of the computer software, the vertical MI lengths were measured from the top of the ball attachment to the MI apex (Fig. 3) and divided by the real length. The Vertical Magnification Factor (VMF) was calculated for every single MI to consider any individual implant inclination in the vestibule-oral direction. Following calibration to the real implant length and with the help of a horizontal ledger line (Fig. 4), the VBL was measured from the upper shoulder of the insertion square to the first marginal bone-to-implant contact.^8^^,^^9^^,^^16^

**Fig. 5:**
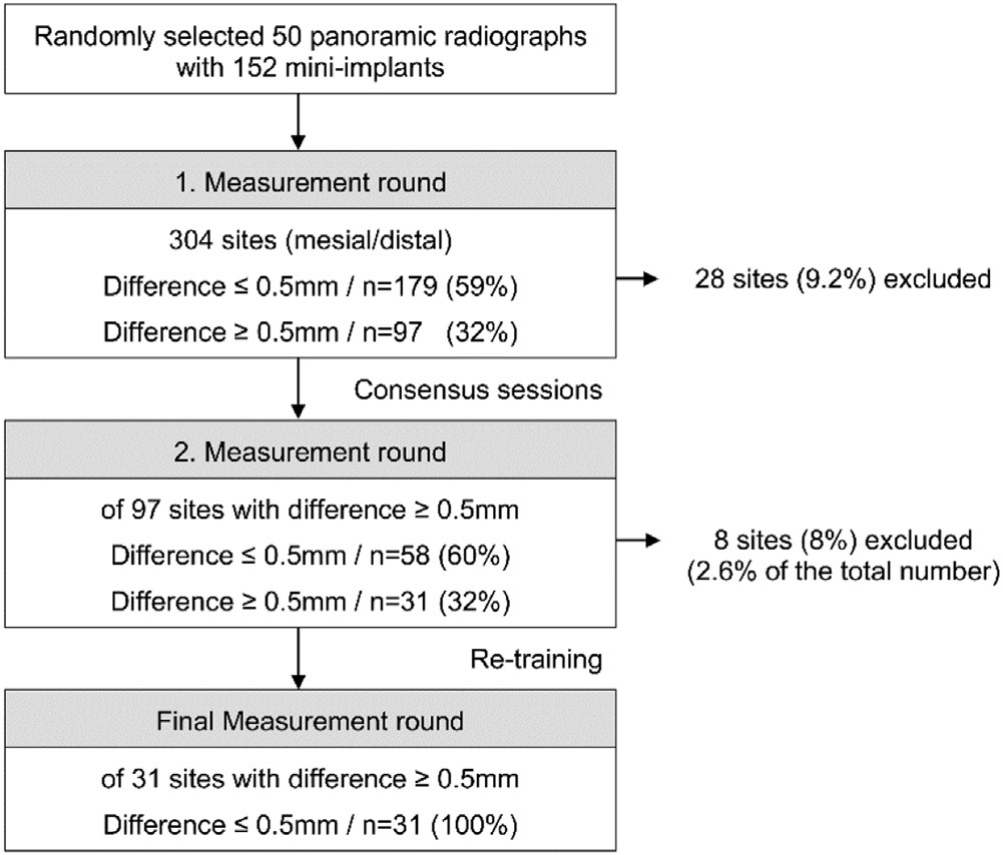
Flowchart of implant site measurements.

This first point of contact is defined as the first visible grey bone pixel closest to the implant identified with the highest zoom factor on the mesial and the distal aspects. Each measurement was projected orthogonally on the central axis. An overview of the study method protocol (implant sites measurements) is shown in a flowchart diagram (Fig. 5). In cases of intra-and inter-rater differences of more than ≥ 0.5 mm, the respective sites were again analyzed in consensus sessions and were either excluded or measured a second time by all examiners.

### 2.3 Statistical methods

**Fig. 6:**
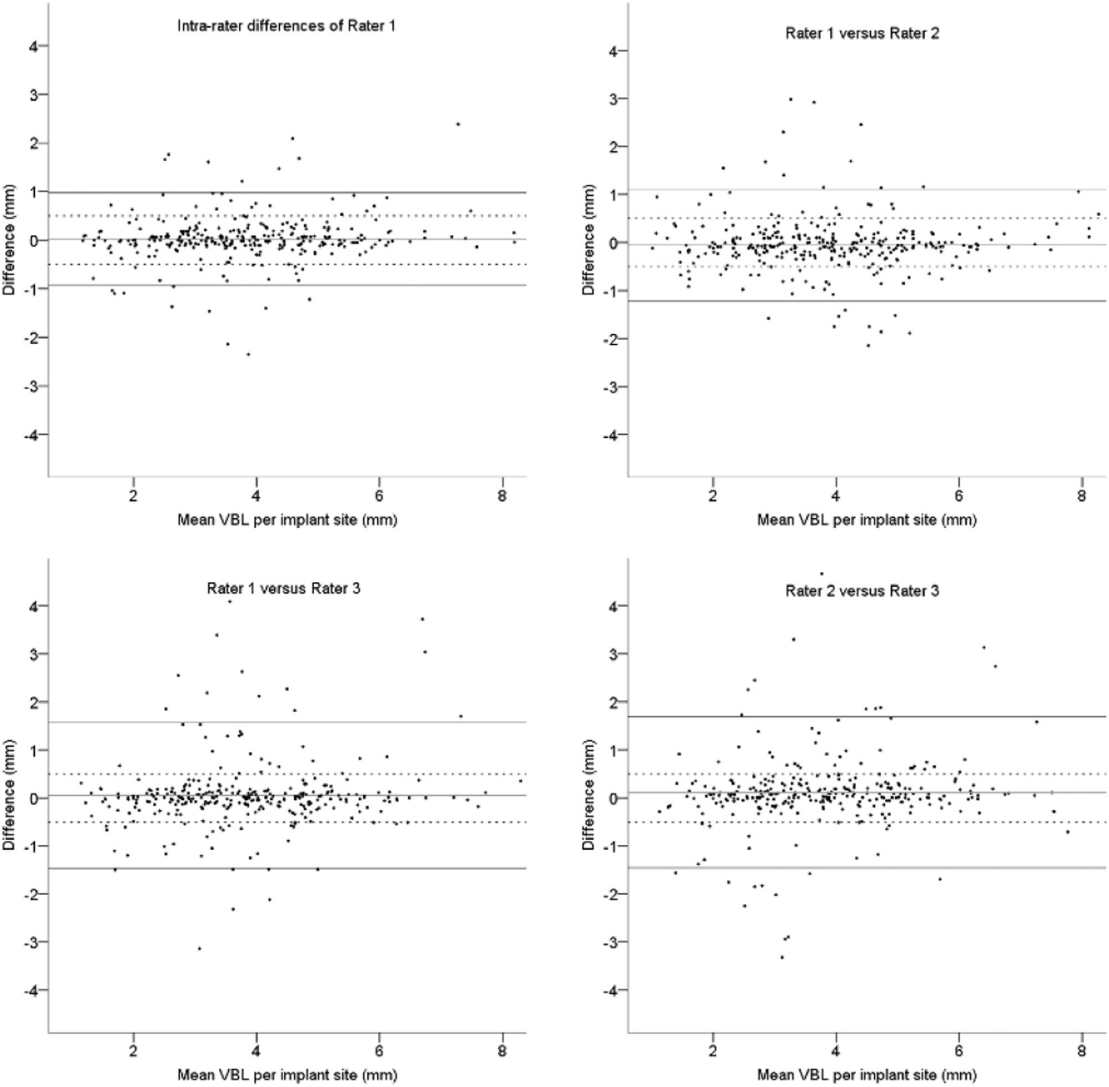
Bland Altman plots of the intra-rater differences of Rater 1 and inter-rater differences for Vertical Bone Level (VBL) values at the first measurement round; solid lines: upper and lower limit of the 95 % Confidence Interval, dashed lines: 0.5 mm limits.

**Fig. 7:**
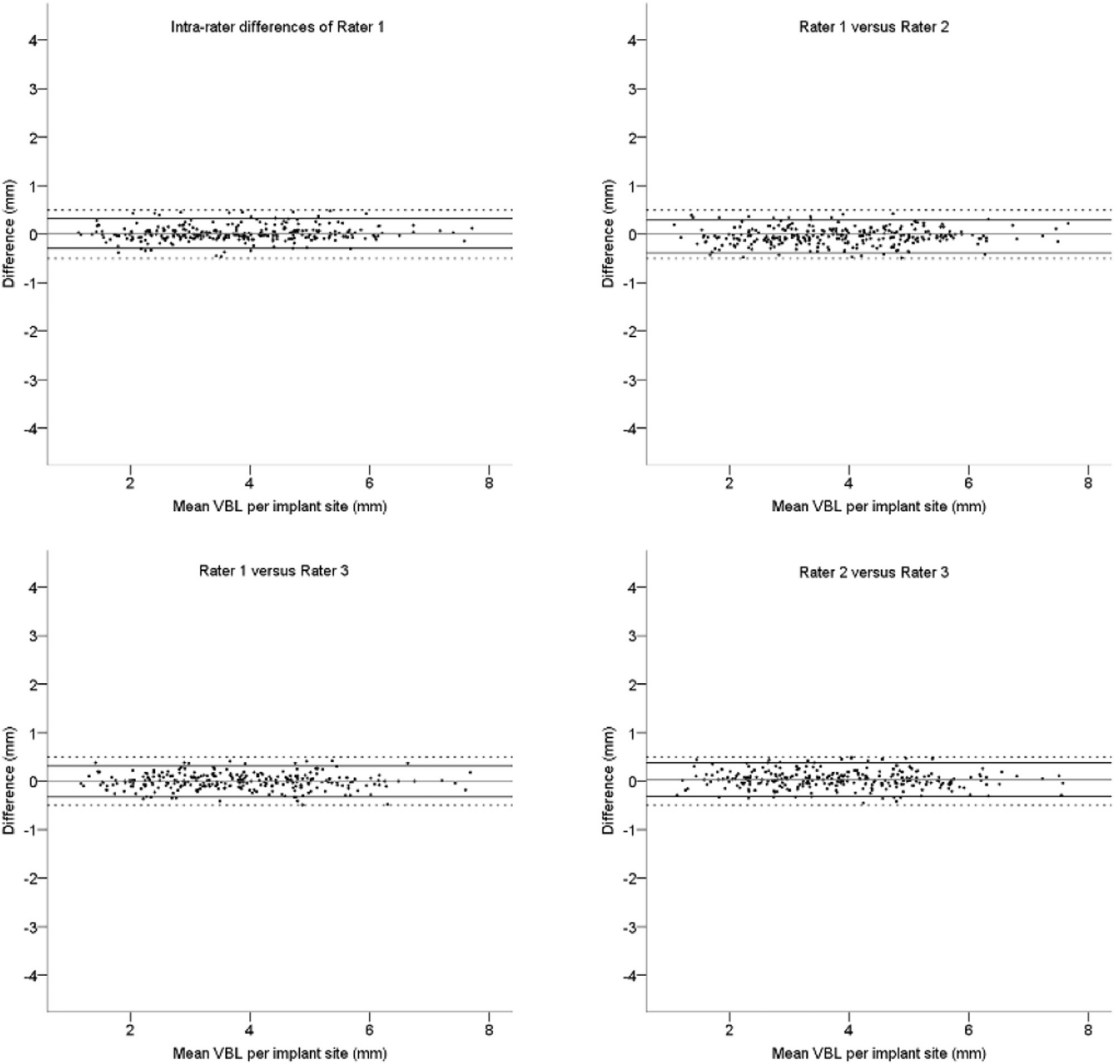
Bland Altman plots of the intra-rater differences of Rater 1 and inter-rater differences for Vertical Bone Level (VBL) values at the final measurement round; solid lines: upper and lower limit of the 95 % Confidence Interval, dashed lines: 0.5 mm limits.

Statistical analyses were performed using SPSS Statistics, version 23, IBM Corporation, and Stata software, release 17.0 (Stata Corporation, College Station, TX, USA). The intra-class correlation coefficient (ICC) was applied to estimate the intra- and inter-rater reliability. Differences in VBL measurements are shown in Bland-Altman plots and compared with clinical relevance (Figs. 6 and 7).

The authors followed de Vet and colleagues and presented the Standard Error of Measurement (SEM; not to be confused with the standard error of the mean) for measures of absolute agreement and consistency (denoted by (2,*k*) and (3,*k*), respectively, a designation used by Koo and colleagues (note that this agreement has a different meaning, it does not necessarily designate an agreement parameter).^18^^,^^29^ The SEM for *k* repeated measurements was divided by √(*k*). The SDC is based on α = 0.05 and defined as 1.96*√(2)* SEM to indicate 95 % credibility of a real change in the true values.^30^ An α level of 0.50 seems well justified for easy interpretation and is calculated as 0.674*√(2)* SEM to indicate 50 % credibility of a real change in the true values.^31^

## 3 Results

**Fig. 8:**
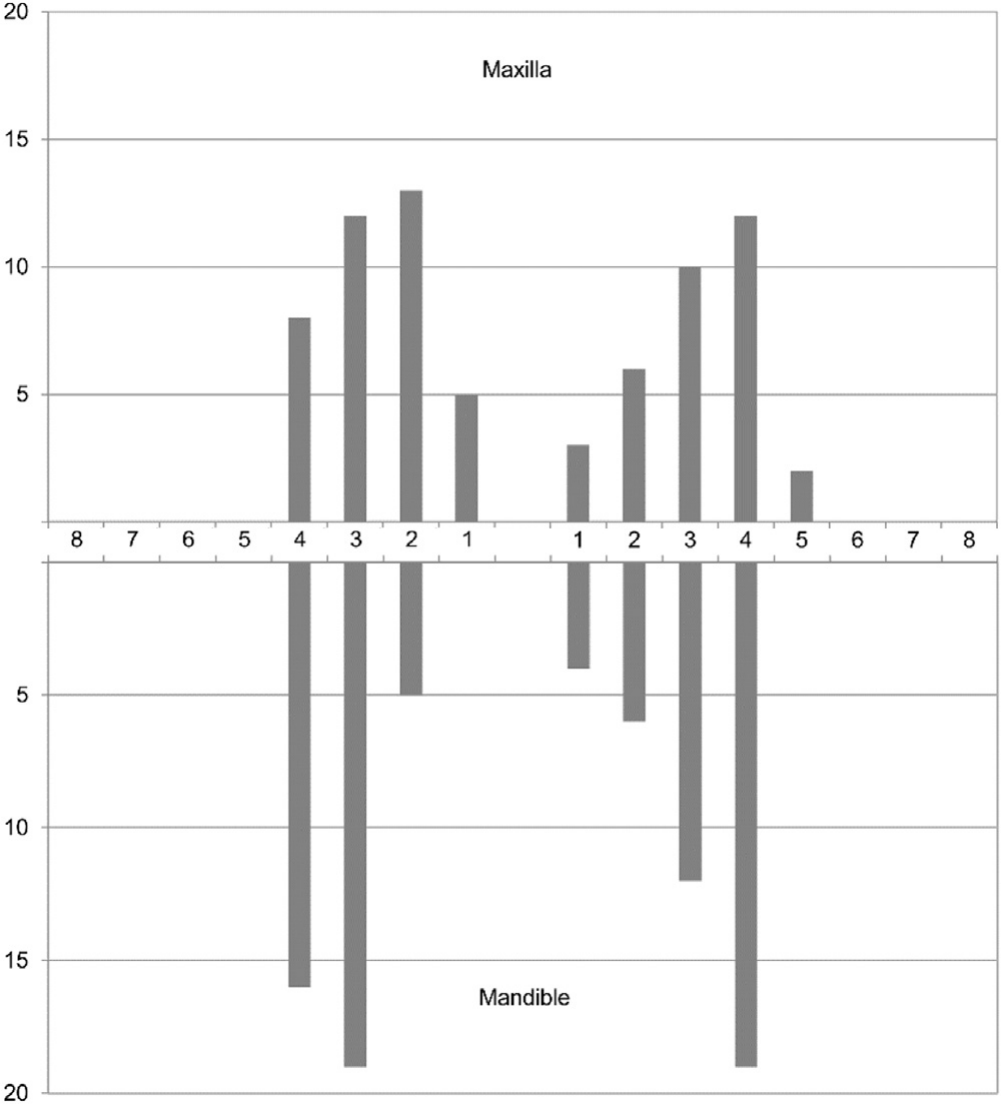
Distribution of mini-implants by tooth site (FDI tooth numbering system).

A total of 71 maxillary MIs and 81 mandibular MIs were evaluated with 50 digital panoramic radiographs. The MIs were placed in regions of incisors (*n* = 42; 28 %), canines (*n* = 53; 35 %), and premolars (*n* = 57; 38 %) (Fig. 8) For all observers similar VMFs of about 1.2 were calculated independently from the placement region with excellent intra- and inter-rater reliability (ICC = 0.999).

The agreement of the first and (corrected) final measurements is demonstrated in Figs. 6 and 7, respectively. In 28 mesial or distal sites (9 %) of the 304 measuring points the VBL could initially not be assessed for the following reasons: (i) Out of focus (edge or motion blurring), (ii) Artefacts by the PR bite block or (iii) Overlapping of anatomical structures. The radiologist rated more sites as unquantifiable than the research assistants (36 vs. 28, respectively). In the first measurement round, 97 (32 %) sites revealed inter-rater differences of 0.5 mm or more (Fig. 6). Following a consensus session among the observers, a second measuring round was performed. As a result, 58 out of the 97 sites showed ≤ 0.5 mm inter-rater differences. The remaining 39 sites were surveyed by a clinically and radiologically experienced supervisor and the observers were trained once again. After a third observation round and the exclusion of 8 unquantifiable sites, all VBL differences were ≤ 0.5 mm (Fig. 7).

For first and final measurements, Tables 1 and 2 present intra- and inter-rater statistics, respectively. In intra-rater statistics, the SEM was improved from 0.26 mm to 0.11 mm, and the SDC was correspondingly improved from 0.73 mm to 0.31 mm (Table 1). The SDC of 0.31 mm indicates no 95 % credibility of a real change in the true values as the SDC is greater than the MCIC of 0.2 mm. The SDC of 0.11 mm, however, indicates 50 % credibility of a real change in the true values as the SDC is less than the MCIC. From the SEM it can be calculated that a SDC of 0.2 indicates 80 % credibility of a real change. Of note, the ICCs for intra- and inter-comparisons were good to excellent already at the first measurement^29^ (Tables 1 and 2).

**Table 1:** Intra-rater statistics for repeated vertical bone level measurements of Rater 1.

	First measurement		Final measurement	
	Single measurement (*k* = 1)	Mean of two multiple measurement (*k* = 2)	Single measurement (*k* = 1)	Mean of two multiple measurements (*k* = 2)
SEM^a^_(2,_*_k_*_)_, mm	0.2647	0.1872	0.1107	0.0783
SEM_(3,_*_k_*_)_, mm	0.2637	0.1865	0.1105	0.0781
SDC^b^_(2,_*_k_*_)_, mm; α = 0.05	0.7338	0.5189	0.3070	0.2171
SDC_(2,_*_k_*_)_, mm; α = 0.50	0.2524	0.1784	0.1056	0.0746
SDC_(3,_*_k_*_)_, mm; α = 0.05	0.7310	0.5169	0.3062	0.2165
SDC_(3,_*_k_*_)_, mm; α = 0.50	0.2514	0.1777	0.1053	0.0744
ICC^c^_(2,_*_k_*_)_ (95 % CI^d^)	0.96 (0.95–0.97)	0.98 (0.97–0.98)	0.99 (0.99–0.99)	1.00 (1.00–1.00)
ICC_(3,_*_k_*_)_ (95 % CI)	0.96 (0.95–0.97)	0.98 (0.97–0.98)	0.99 (0.99–0.99)	1.00 (1.00–1.00)

(2,k) Absolute agreement.

(3,k) Consistency.

a Standard error of measurement.

b Smallest detectable change.

c Intraclass correlation coefficient.

d Confidence interval.

**Table 2:** The inter-rater statistics for vertical bone level measurements of Raters 1 to 3.

	First measurement		Final measurement	
	Single measurement (*k* = 1)	Mean of two multiple measurement (*k* = 2)	Single measurement (*k* = 1)	Mean of two multiple measurements (*k* = 2)
SEM^a^_(2,_*_k_*_)_, mm	0.5505	0.3178	0.1235	0.0713
SEM_(3,_*_k_*_)_, mm	0.5491	0.3170	0.1216	0.0702
SDC^b^_(2,_*_k_*_)_, mm; α = 0.05	1.5259	0.8810	0.3422	0.1976
SDC_(2,_*_k_*_)_, mm; α = 0.50	0.5247	0.3029	0.1177	0.0679
SDC_(3,_*_k_*_)_, mm; α = 0.05	1.5220	0.8788	0.3371	0.1946
SDC_(3,_*_k_*_)_, mm; α = 0.50	0.5234	0.3022	0.1159	0.0669
ICC^c^_(2,_*_k_*_)_ (95 % CI^d^)	0.84 (0.81–0.87)	0.94 (0.93–0.95)	0.99 (0.99–0.99)	1.00 (1.00–1.00)
ICC_(3,_*_k_*_)_ (95 % CI)	0.84 (0.81–0.87)	0.94 (0.93–0.95)	0.99 (0.99‒0.99)	1.00 (1.00–1.00)

(2,k) Absolute agreement.

(3,k) Consistency.

a Standard error of measurement.

b Smallest detectable change.

c Intraclass correlation coefficient.

d Confidence interval.

## 4 Discussion

Regardless of excellent ICCs, this experimental study shows marked discrepancies of more than one millimeter in the initial assessment of VBL values on PRs around MIs after repeated measurements of one observer and between the three observers even though poor radiographs were initially not considered. After re-calibration sessions and the exclusion of further unquantifiable radiographs, all intra- and inter-rater differences could be diminished to ≤ 0.5 mm. Thus, the agreement parameters, i.e., SEM and SDC, after the final measurement round were in the range of the MCIC of 0.2 mm.

This study has some limitations. First, PR was used in this study although IRs are the gold standard of two-dimensional imaging of VBL at implants.^4^ However, digital PR has the potential to be as reliable as IR for VBL measurements at implants.^16^ Second, the validity of the measurements could not be verified. Validation is only possible intraoperatively in patients, and experimental in human cadavers or animals.^11^^,^^13^^,^^17^^,^^32^ Most validity studies used IR and showed an overestimation of the radiographic VBL (or underestimation of peri‑implant bone defects) between 0.5 and 2.5 mm compared to the true values.^11^, ^12^, ^13^ The deeper the vertical bone defects, the higher the differences between radiographic and intraoperative VBL values.^12^ The VBL overestimation could be explained by the partial resorption with retention of the cortical plate in intra-bony defects. As such, the bone-to-implant border is difficult to detect in two-dimensional radiographs.^13^ Also, contrary to PR and IR, conventional or Cone-Beam Computed Tomography (CBCT) can show buccal and lingual sites of the implants without noteworthy magnification. However, high cost, increased exposure to radiation, and the presence of metal artifacts are considered the main limitations of these techniques.^33^ Surprisingly, two-wall bone defects affecting the oral and buccal part of an implant were more often assessed correctly by PR than by IR or CBCT in a human cadaver study.^17^ Third, a total of 36 uninterpretable implant sites decreased the overall number of 304 measuring points by 11.8 %. Nevertheless, the number of remaining MIs should be sufficient to verify possible bone level changes. In other studies, a considerable number of PR images (8 %‒39 %) were also excluded due to poor quality.^23^^,^^24^^,^^34^ Exclusion reasons were low density, low contrast, vague bone borders, noise, or blurred implants which are out of focus. In a study, one-fourth of 1782 PR images were categorized as diagnostically unacceptable because of patient malpositioning, head movement, or other unnamed reasons.^35^ The proportions of excluded IR images in similar studies were 5 %^23^ and 16 %.^13^ Fourth, the examiners had to perform three evaluation rounds until the inter-rater agreement was satisfactory. A consensus session among observers after the first round and further training by an experienced supervisor after the second round could markedly improve the outcome. Hence, in cases of wide variations, the interpretation of VBLs on PRs should be re-examined carefully.^14^ Therefore, the present approach is rather for research purposes and less suitable for clinical application in a dentist's office. The question arises whether it is a limitation or a strength. A similar approach of calibration and training of the examiner was used for better reproducibility in periodontal probing measurements.^36^ Before the main study started the probing data of the training period were monitored and retraining took place if needed to eliminate bias. The training goal was to achieve a minimum level of intra-rater agreement after repeated probing, i.e., 95 % of the differences should be within ±1 mm. Thereafter, a gold standard examiner with the best intra-rater agreement was selected. The difference limit in the present study was set at 0.5 mm for several reasons. The mean inter-rater differences in other studies of ≥ 0.3 mm with standard deviations of ≥ 0.4 mm could be traceable to high proportions of marked discrepancies (≥ 0.5 mm) between raters as seen in other studies.^21^^,^^22^^,^^37^ In one of the first reliability studies 7 % of the VBL differences between two raters were outside the freely chosen tolerance limit of one millimeter.^38^ In other studies, the proportion of > 1 mm inter-rater differences was even 25 %^25^ or 18.8 %.^37^ In one other study with six raters the proportion of VBL differences of ≥ 0.5 mm ranged between 16 % and 35 %.^26^ The strengths of this study are the three examiners with different experiences, among them a radiologist. The PRs for this study were randomly chosen from one of four centers. Thus, not only the measurement conditions but also the X-ray unit and the radiographers were identical. Since every single implant was length-calibrated, both the PR magnification and the implant angulation within the alveolar bone were considered.^10^^,^^39^ Altogether, this is the first evaluation of intra- and inter-rater agreement parameters such as SDC or SEM to estimate the relation to the MCIC.

In fact, radiographic VBLs after the first year are not annually monitored but rather every 3 to 10 years.^1^^,^^2^^,^^5^^,^^21^^,^^34^ Therefore, progressive bone loss, i.e. peri‑implantitis, is commonly larger than 0.2 mm as it is detected as a sum of several years. The literature recommends taking IR additionally if visual assessment of PR is limited, but a precision of ≤ 0.2 mm in IR cannot be obtained.^6^^,^^7^^,^^9^^,^^15^^,^^17^^,^^32^ Contrary to the present study almost all intra- and inter-rater reliability studies of radiographic VBL assessments around implants did not perform further training sessions or re-calibrations between several evaluation rounds.^6^^,^^9^^,^^13^, ^14^, ^15^, ^16^^,^^22^^,^^27^^,^^37^^,^^38^ In order to reduce the rater variability, researchers recommended at least a consensus to determine the exact landmarks for measurements or to identify outliers of one observer by multiple readings.^26^^,^^37^

The discrepancy between excellent ICCs as a reliability measure and multiple intra- and inter-rater differences of more than 0.5 mm VBL of the first evaluation round are confirmed by other researchers.^21^^,^^22^^,^^25^^,^^26^ It is to assume that excellent ICCs do not compulsively imply inter-rater SDCs lower than the MCIC. The differences seemed acceptable in terms of the mean reproducibility. However, for a number of implant sites, there were clinically relevant differences that could affect therapy decisions or research outcomes. To prevent any rater bias in VBL evaluations, the need for more training and more than one observer including agreement sessions is evident.

To determine whether a VBL change in longitudinal observations on an individual implant level is clinically important and not just a measurement error, the SDC must not exceed the MCIC.^19^^,^^39^ In the present inter-rater statistics, the SDC with 95 % credibility could be cut from 0.7 mm in the first evaluation round to 0.3 mm in the final round and was still slightly larger than the MCIC of 0.2 mm. However, in this particular setting, 50 % credibility was added for ease of interpretation – the true SDC should be as likely to be smaller as larger than the SDC with 50 % credibility, and there is no treatment decision to be made based on the SDC.^31^ On that condition, the SDCs of about 0.1 mm both for intra- and inter-rater statistics were clearly lower than the MCIC in the final measurement round. That implies an acceptable level of agreement. Comparisons with previous research are currently impossible failing similar statistical evaluations. It is for future research to judge the SDC not only for PR but also for IR. Observer training and re-calibration should be considered for that purpose. The higher exclusion rate of unreadable implant sites on PR compared to IR should be considered. It is reasonable to assume that the results of the present study can be transferred to evaluations using conventional dental implants.

## 5 Conclusion

The results of this experimental study show that the digital PR can be reliably utilized to determine the VBL around MIs when at least two trained observers are involved, mutual calibration sessions are implemented, and unquantifiable radiographs are excluded.

## Disclaimer statement

Study results have not been presented at conferences or symposia or published.

## Declaration of Competing Interest

The authors declare no conflicts of interest.
